# Supplementary material for: α-L-Fucosidases from an Alpaca Faeces Metagenome: Characterisation of Hydrolytic and Transfucosylation Potential
Source: Int J Mol Sci. 2024 Jan 9;25(2):809. doi: 10.3390/ijms25020809 (PMC10815079; doi:10.3390/ijms25020809)
Supplement: Supplementary file 1 [file ijms-25-00809-s001.zip › Supplementary_file_revised.pdf]

Supplementary materials

α-L-Fucosidases from an Alpaca Faeces Metagenome: Characterisation of Hydrolytic and Transfucosylation Potential

Agnė Krupinskaitė *, Rūta Stanislauskienė, Pijus Serapinas, Rasa Rutkienė, Renata Gasparavičiūtė, Rolandas Meškys and Jonita Stankevičiūtė *

Institute of Biochemistry, Life Sciences Center, Vilnius University, Sauletekio av. 7, LT-10257 Vilnius, Lithuania
*Correspondence: agne.krupinskaite@gmc.vu.lt (A.K.); jonita.stankeviciute@bchi.vu.lt (J.S.)

Table S1 Specific primers for the amplification of the α-L-fucosidases. The bold part of the primers complements the pLATE31 vector.

Gene	Primer name	Primer sequence
fuc25A	fuc25A F	**AGAAGGAGATATAACT**ATGAAAATGCTTAAACCCGAAAAGAG
	fuc25A R	**GTGGTGGTGATGGTGATGGCC**GGCAAGAGTAACCTTTGCAACTC
fuc25C	fuc25C F	**AGAAGGAGATATAACT**ATGGATAAAAAAGCATATTTAAAAACAATCG
	fuc25C R	**GTGGTGGTGATGGTGATGGCC**GTTTGTAATAACCTTCACTACCACC
fuc25D	fuc25D F	**AGAAGGAGATATAACT**ATGAAAGGTAACAATATTATG
	fuc25D R	**GTGGTGGTGATGGTGATGGCCTTTAACCTTTATCTCTGCAAC**
fuc25E	fuc25E F	**AGAAGGAGATATAACTATGAGTATTCCCACCCCGCTG**
	fuc25E R	**GTGGTGGTGATGGTGATGGCCGCGGGTTTCGATCTTCAGG**

Table S2 Identity comparison of α-L-fucosidases from alpaca faeces metagenome. Identity is expressed in percentage value using the Clustal Omega algorithm.

**α-L-Fucosidase**	**Enzyme identity, %**			
	**Fuc25A**	**Fuc25C**	**Fuc25D**	**Fuc25E**
Fuc25A	100	29.09	53.94	53.59
Fuc25C	29.09	100	24.94	27.23
Fuc25D	53.94	24.94	100	49.15
Fuc25E	53.59	27.23	49.15	100

Table S3 Transfucosylation results when reactions are performed under normalised protein concentration evaluated by HPLC-MS. If the peak area of the transfucosylation reaction sample was divided by the negative control peak area and the difference was <3, the transfucosylation reaction is happening at the trace level. If transfucosylation reaction is not observed, it is indicated with (-).

**Acceptor compound**	**Fuc25A**	**Fuc25D**	**Fuc25C**	**Fuc25E**	**Observed mass, m/z**
	Monosaccharides				
D-Glucose	+	+	+	+	365 (+): D-Glucose + L-fucose + K^+ – H_2O
D-Galactose	+	+	+	+	365 (+): D-Galactose + L-fucose + K^+ – H_2O
D-Fructose	+	+	+	+	365 (+): D-Fructose + L-fucose + K^+ – H_2O
L-Fucose	Traces*	Traces*	Traces*	+	348.90 (+): L-Fucose + L-fucose + K^+ – H_2O
N-Acetylglucosamine	+	+	+	+	405.90 (+): N-Acetylglucosamine + L-fucose + K^+ – H_2O
L-Rhamnose	+	+	Traces*	+	349.00 (+): L-Rhamnose + L-fucose + K^+ – H_2O
D-Ribose	+	+	+	+	334.90 (+): D-Ribose + L-fucose + K^+ – H_2O
D-Xylose	+	+	+	+	335.80 (+): D-Xylose + L-fucose + K^+ – H_2O
D-Mannose	+	+	+	+	364.85 (+): D-Mannose + L-fucose + K^+ – H_2O
	Disaccharides				
Lactose	+	+	+	+	526.85 (+): Lactose + L-fucose + K^+ – H_2O
Maltose	+	+	+	+	527.00 (+): Maltose + L-fucose + K^+ – H_2O
	Amino acids				
L-Serine	+	+	+	+	290.00 (+): L-Serine + L-fucose + K^+ – H_2O
L-Threonine	+	+	+	+	304.00 (+): L-Threonine + L-fucose + K^+ – H_2O
D-Serine	+	+	+	+	290.00 (+): D-Serine + L-fucose + K^+ – H_2O
D-Threonine	+	+	+	+	304.00 (+): D-Threonine + L-fucose + K^+ – H_2O

Table S4 Transfucosylation results when reactions are performed under normalised protein concentration evaluated by HPLC-MS. The numbers in the enzyme column are the percentage values of the observed transfucoylatation reaction product. The highest value observed with a specific acceptor substrate is evaluated as 100 %; other results are evaluated respectively to the value that was observed for the 100 %. If the peak area of the transfucosylation reaction sample was divided by the negative control peak area and the difference was <3, the transfucosylation reaction is happening at the trace level. If transfucosylation reaction is not observed, it is indicated with (-).

Acceptor compound	Degree of polymerisation	Transfucosylation results, %				Observed mass, m/z
		Fuc25A	Fuc25D	Fuc25C	Fuc25E	
D-Glucose	DP1	100	43	12	75	365 (+): D-Glucose + L-fucose + K^+ – H_2O
D-Xylose	DP1	100	74	17	82	336 (+): D-Xylose + L-fucose + K^+ – H_2O
D-Trehalose	DP2	100	89	26	61	527 (+): D-Trehalose + L-fucose + K^+ – H_2O
Raffinose	DP3	100	11	Traces	21	690 (+): Raffinose + L-fucose + K^+ – H_2O
Maltotriose	DP3	100	32	Traces	61	689 (+): Maltotriose + L-fucose + K^+ – H_2O
Xylooligosacharides	DP1	-	-	-	-	335 (+): L-Xylose + L-fucose + K^+ – H_2O
	DP2	+*	+*	Traces	+*	467 (+): L-Xylose disaccharide + L-fucose + K^+ – H_2O
	DP3	+*	+	Traces	+*	599 (+): L-Xylose trisaccharide + L-fucose + K^+ – H_2O
	DP4	+*	Traces	Traces	+*	731 (+): L-Xylose tetra saccharide + L-fucose + K^+ – H_2O
	DP5	Traces	Traces	-	Traces	863 (+): L-Xylose penta saccharide + L-fucose + K^+ – H_2O
	DP6	Traces	+*	+*	Traces	995 (+): L-Xylose hexa saccharide + L-fucose + K^+ – H_2O
Galactooligosaccharides	DP1	Traces	Traces	Traces	Traces	365 (+): D-Galactose+ L-fucose + K^+ – H_2O
	DP2	Traces	Traces	Traces	Traces	527 (+): DP2 + L-fucose + K^+ – H_2O
	DP3	Traces	Traces	Traces	Traces	689 (+): DP3 + L-fucose + K^+– H_2O
	DP4	Traces	Traces	Traces	Traces	851 (+): DP4 + L-fucose + K^+ – H_2O
	DP5	Traces	Traces	+	Traces	1013 (+): DP5 + L-fucose + K^+ – H_2O

*The percentage evaluation is not applied to compound mixtures such as xylooligosaccharides and galactooligosaccharides; if reaction products are observed, this is indicated with the symbol (+).

Table S5 HPLC-MS analysis of the reaction mixtures sampled after transfucosylation of saccharides and amino acids. Extracted ion chromatograms of fucosylation products of (A) xylooligosaccharide: DP1, DP2, DP3, DP4, DP5, DP6; (B) galactooligosaccharide: DP1, DP2, DP3, DP4, DP5; (C) D-Xylose; (D) D-Glucose; (E) D-Trehalose; (F) D-Raffinose; (G) Maltotriose; (H) D-Fructose; (I) L-Fucose; (J) Maltotriose; (K) D-Ribose; (L) D-serine; (M) D-Threonine; (N) L-Serine (O) L-Threonine; (P) D-Galactose; (Q) D-Glucose; (R) Lactose; (S) Maltose; (T) D-Mannose; (U) N-Acetyl glucosamine; (V) L-Rhamnose; (Z) D-Xylose

<table>
<tr><td rowspan="2">Acceptor molecule</td><td colspan="5">Enzyme used for reaction and mass spectra of fucosylated compounds</td></tr>
<tr><td>Negative control</td><td>Fuc25A</td><td>Fuc25D</td><td>Fuc25E</td><td>Fuc25C</td></tr>
<tr><td>(A) Xylooligosaccharide (normalized)</td>
<td>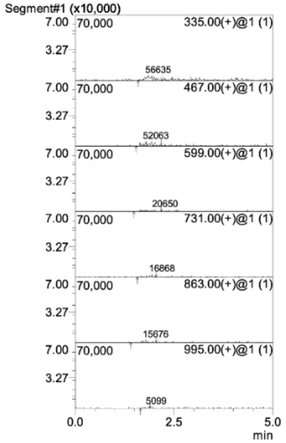
DP1 [M+K+]+=335
DP2 [M+K+]+=467
DP3 [M+K+]+=599
DP4 [M+K+]+=731
DP5 [M+K+]+=863
DP6 [M+K+]+=995</td>
<td>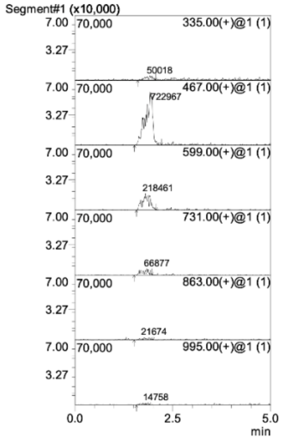
DP1 [M+K+]+=335
DP2 [M+K+]+=467
DP3 [M+K+]+=599
DP4 [M+K+]+=731
DP5 [M+K+]+=863
DP6 [M+K+]+=995</td>
<td>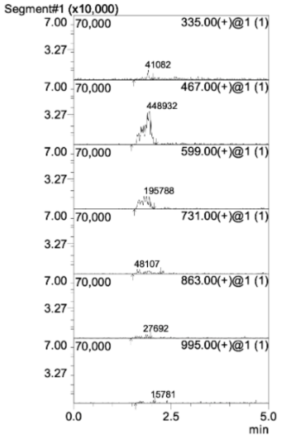
DP1 [M+K+]+=335
DP2 [M+K+]+=467
DP3 [M+K+]+=599
DP4 [M+K+]+=731
DP5 [M+K+]+=863
DP6 [M+K+]+=995</td>
<td>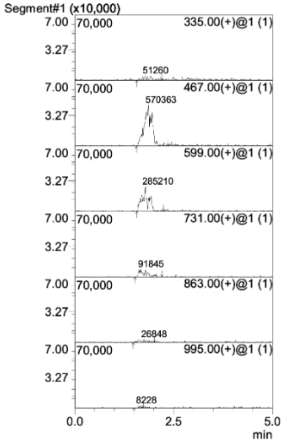
DP1 [M+K+]+=335
DP2 [M+K+]+=467
DP3 [M+K+]+=599
DP4 [M+K+]+=731
DP5 [M+K+]+=863
DP6 [M+K+]+=995</td>
<td>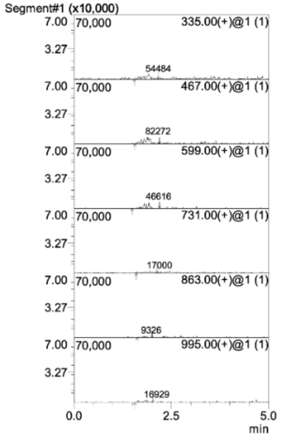
DP1 [M+K+]+=335
DP2 [M+K+]+=467
DP3 [M+K+]+=599
DP4 [M+K+]+=731
DP5 [M+K+]+=863
DP6 [M+K+]+=995</td></tr>
</table>

Acceptor molecule	Enzyme used for reaction and mass spectra of fucosylated compounds				
	Negative control	Fuc25A	Fuc25D	Fuc25E	Fuc25C
(B) Galactooligosaccharide (normalized)	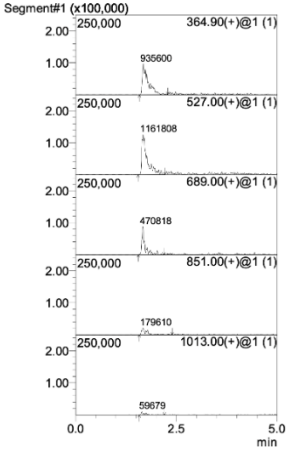 DP1 [M+K+]+=365<br>DP2 [M+K+]+=527<br>DP3 [M+K+]+=689<br>DP4 [M+K+]+=851<br>DP5 [M+K+]+=1013	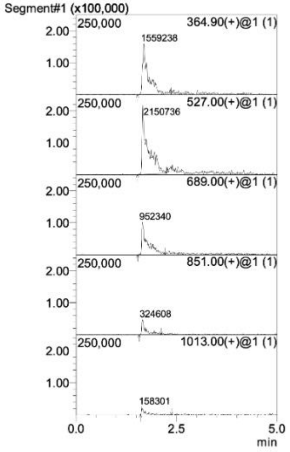 DP1 [M+K+]+=365<br>DP2 [M+K+]+=527<br>DP3 [M+K+]+=689<br>DP4 [M+K+]+=851<br>DP5 [M+K+]+=1013	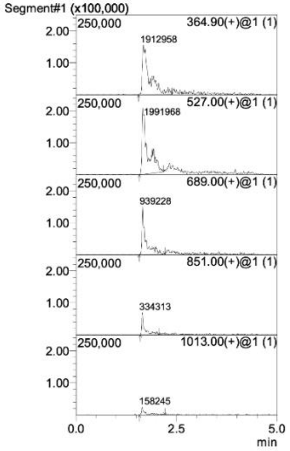 DP1 [M+K+]+=365<br>DP2 [M+K+]+=527<br>DP3 [M+K+]+=689<br>DP4 [M+K+]+=851<br>DP5 [M+K+]+=1013	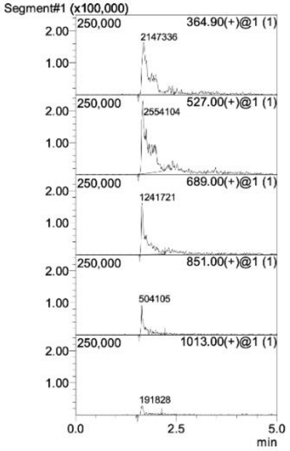 DP1 [M+K+]+=365<br>DP2 [M+K+]+=527<br>DP3 [M+K+]+=689<br>DP4 [M+K+]+=851<br>DP5 [M+K+]+=1013	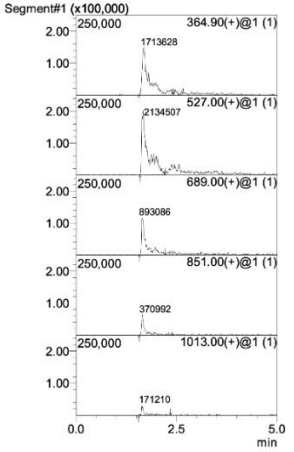 DP1 [M+K+]+=365<br>DP2 [M+K+]+=527<br>DP3 [M+K+]+=689<br>DP4 [M+K+]+=851<br>DP5 [M+K+]+=1013
(C) D-Xylose (normalized)	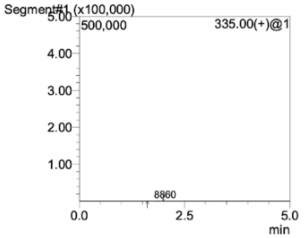 [M+K+]+=335	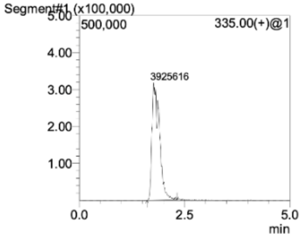 [M+K+]+=335	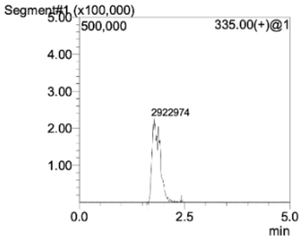 [M+K+]+=335	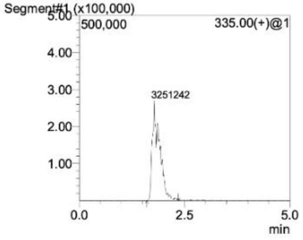 [M+K+]+=335	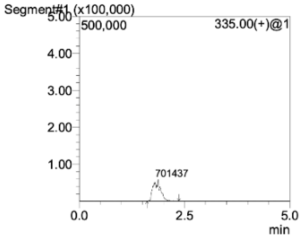 [M+K+]+=335

Acceptor molecule	Enzyme used for reaction and mass spectra of fucosylated compounds				
	Negative control	Fuc25A	Fuc25D	Fuc25E	Fuc25C
(D) D-Glucose (normalized)	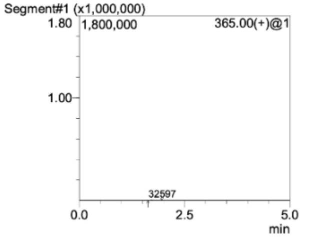 [M+K⁺]⁺=365	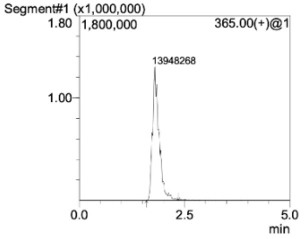 [M+K⁺]⁺=365	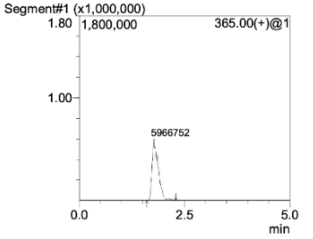 [M+K⁺]⁺=365	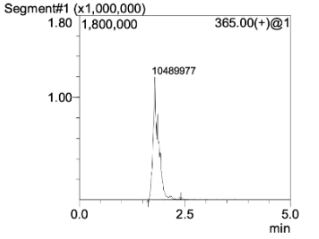 [M+K⁺]⁺=365	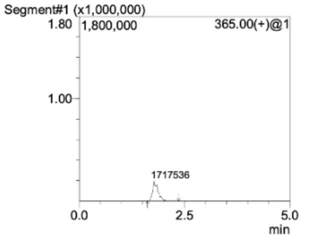 [M+K⁺]⁺=365
(E) D-Trehalose (normalized)	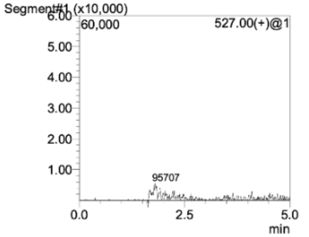 [M+K⁺]⁺=527	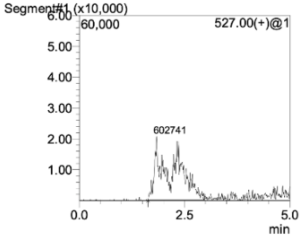 [M+K⁺]⁺=527	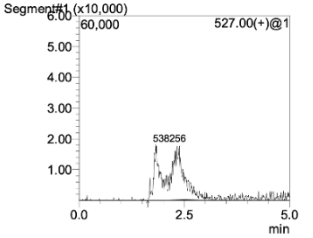 [M+K⁺]⁺=527	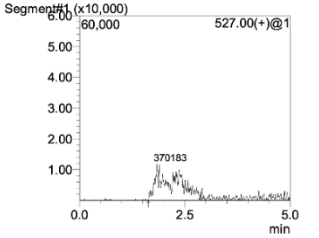 [M+K⁺]⁺=527	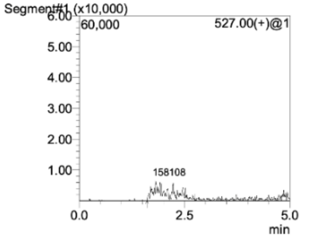 [M+K⁺]⁺=527
(F) D-Raffinose (normalized)	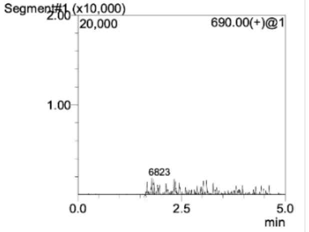 [M+K⁺]⁺=690	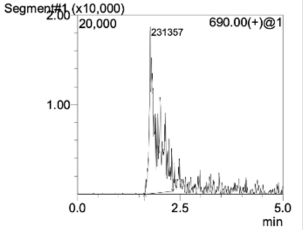 [M+K⁺]⁺=690	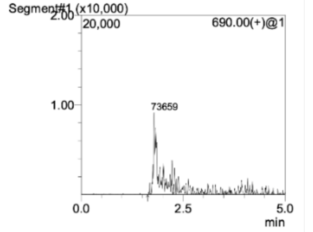 [M+K⁺]⁺=690	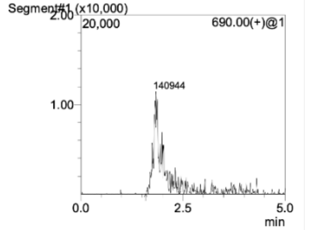 [M+K⁺]⁺=690	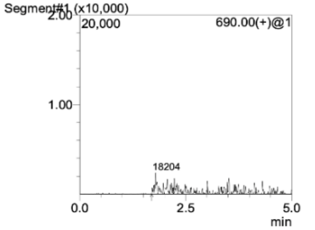 [M+K⁺]⁺=690
(G) Maltotriose (normalized)	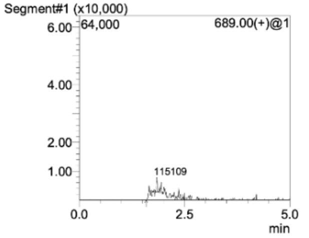 [M+K⁺]⁺=689	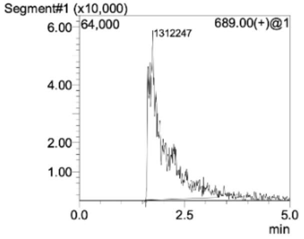 [M+K⁺]⁺=689	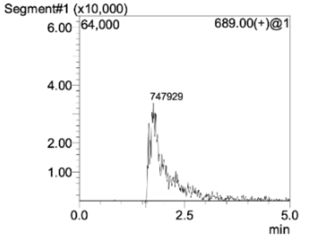 [M+K⁺]⁺=689	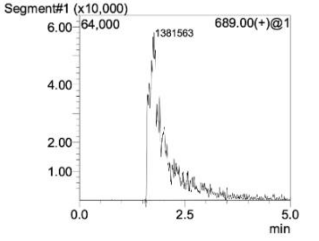 [M+K⁺]⁺=689	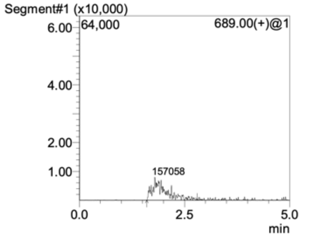 [M+K⁺]⁺=689

<table>
<tr><th rowspan="2">Acceptor molecule</th><th colspan="5">Enzyme used for reaction and mass spectra of fucosylated compounds</th></tr>
<tr><th>Negative control</th><th>Fuc25A</th><th>Fuc25D</th><th>Fuc25E</th><th>Fuc25C</th></tr>
<tr><td>(H)
D-Fructose</td>
<td>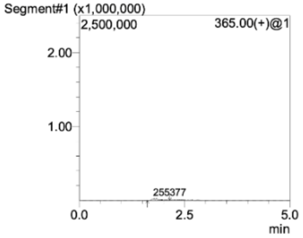
[M+K⁺]⁺=365</td>
<td>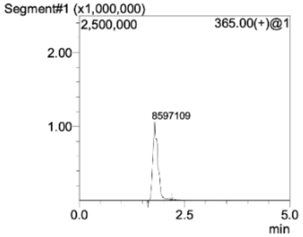
[M+K⁺]⁺=365</td>
<td>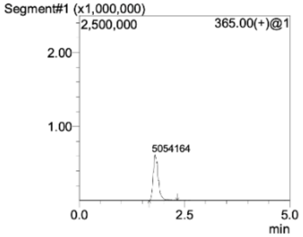
[M+K⁺]⁺=365</td>
<td>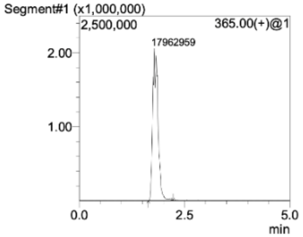
[M+K⁺]⁺=365</td>
<td>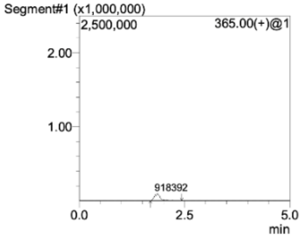
[M+K⁺]⁺=365</td></tr>
<tr><td>(I)
L-Fucose</td>
<td>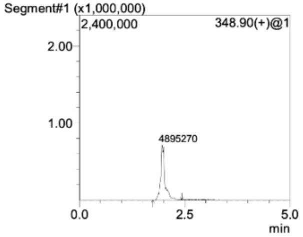
[M+K⁺]⁺=349</td>
<td>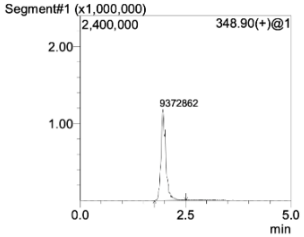
[M+K⁺]⁺=349</td>
<td>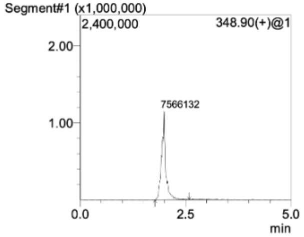
[M+K⁺]⁺=349</td>
<td>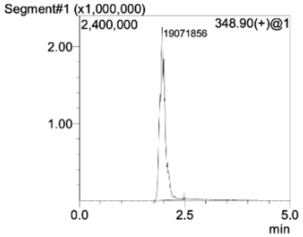
[M+K⁺]⁺=349</td>
<td>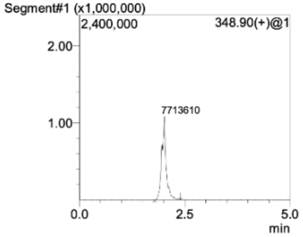
[M+K⁺]⁺=349</td></tr>
<tr><td>(J)
Maltotriose</td>
<td>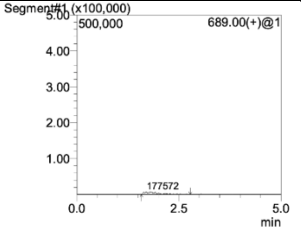
[M+K⁺]⁺=689</td>
<td>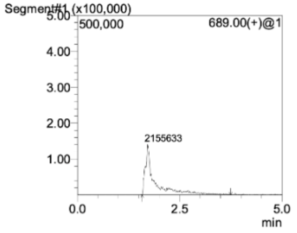
[M+K⁺]⁺=689</td>
<td>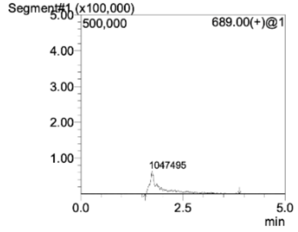
[M+K⁺]⁺=689</td>
<td>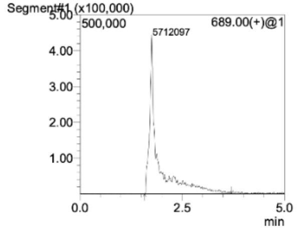
[M+K⁺]⁺=689</td>
<td>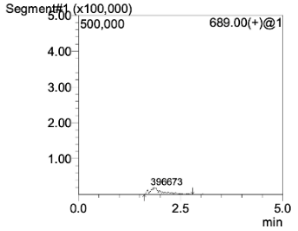
[M+K⁺]⁺=689</td></tr>
<tr><td>(L)
D-Serine</td>
<td>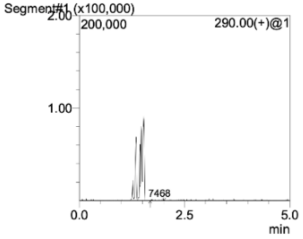
[M+K⁺]⁺=290</td>
<td>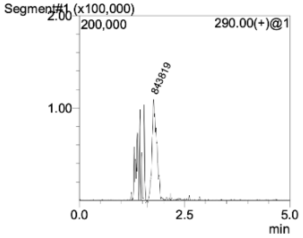
[M+K⁺]⁺=290</td>
<td>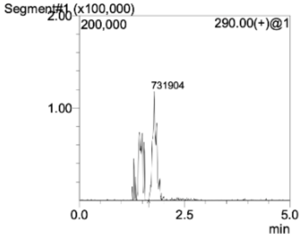
[M+K⁺]⁺=290</td>
<td>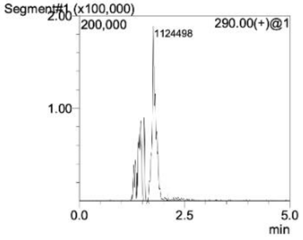
[M+K⁺]⁺=290</td>
<td>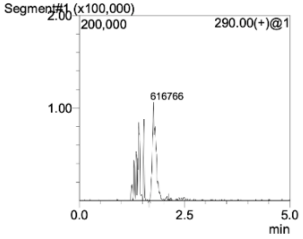
[M+K⁺]⁺=290</td></tr>
</table>

Acceptor molecule	Enzyme used for reaction and mass spectra of fucosylated compounds				
	Negative control	Fuc25A	Fuc25D	Fuc25E	Fuc25C
(M)<br>D-Threonine	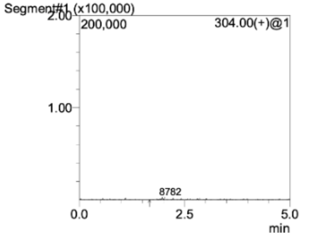<br>$[M+K^+]^+=304$	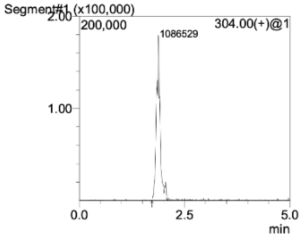<br>$[M+K^+]^+=304$	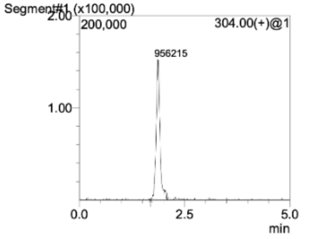<br>$[M+K^+]^+=304$	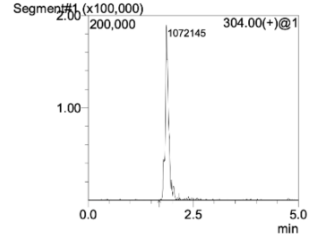<br>$[M+K^+]^+=304$	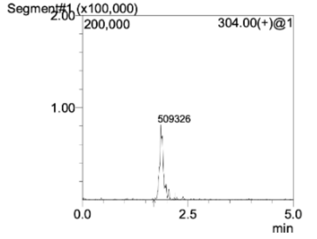<br>$[M+K^+]^+=304$
(N)<br>L-Serine	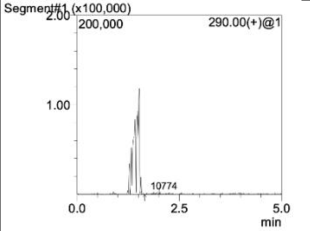<br>$[M+K^+]^+=290$	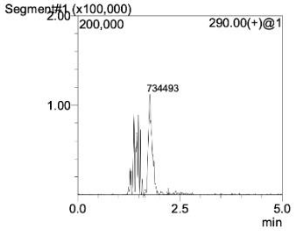<br>$[M+K^+]^+=290$	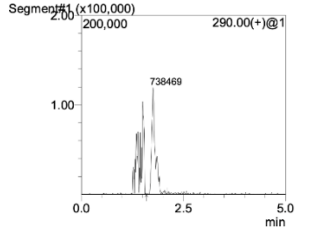<br>$[M+K^+]^+=290$	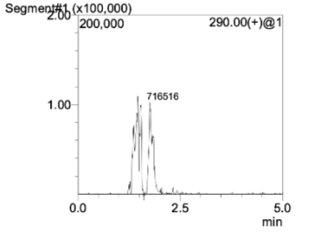<br>$[M+K^+]^+=290$	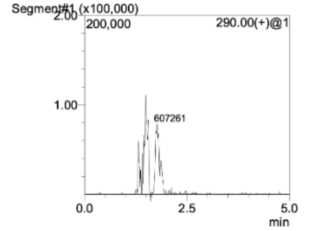<br>$[M+K^+]^+=290$
(O)<br>L-Threonine	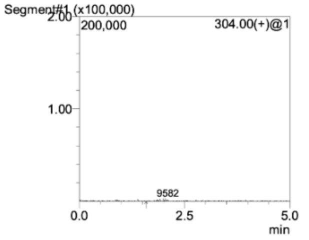<br>$[M+K^+]^+=304$	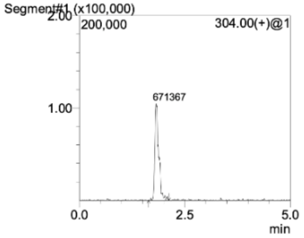<br>$[M+K^+]^+=304$	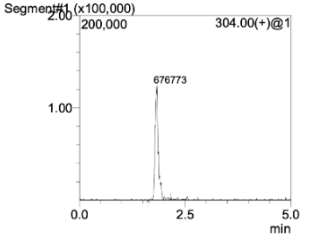<br>$[M+K^+]^+=304$	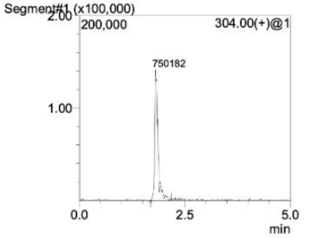<br>$[M+K^+]^+=304$	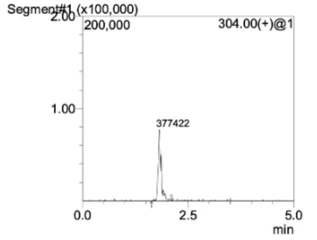<br>$[M+K^+]^+=304$
(P)<br>D-Galactose	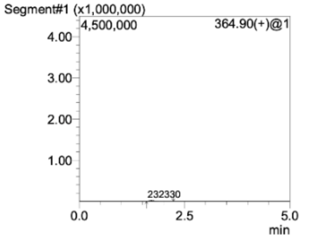<br>$[M+K^+]^+=365$	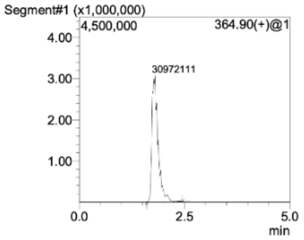<br>$[M+K^+]^+=365$	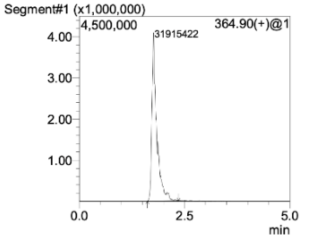<br>$[M+K^+]^+=365$	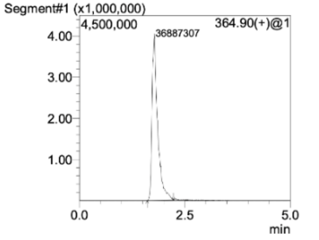<br>$[M+K^+]^+=365$	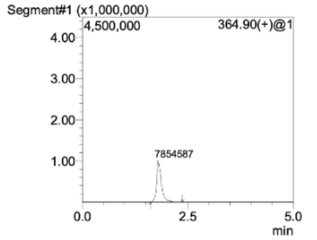<br>$[M+K^+]^+=365$

Acceptor molecule	Enzyme used for reaction and mass spectra of fucosylated compounds				
	Negative control	Fuc25A	Fuc25D	Fuc25E	Fuc25C
(Q)<br>D-Glucose	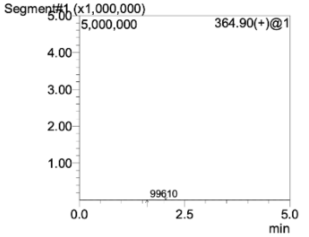<br>$[M+K^+]^+=365$	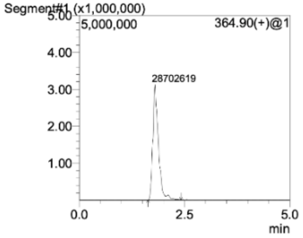<br>$[M+K^+]^+=365$	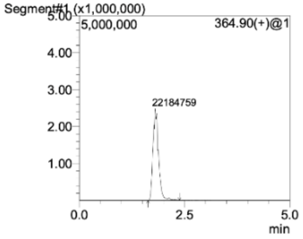<br>$[M+K^+]^+=365$	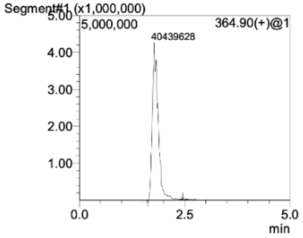<br>$[M+K^+]^+=365$	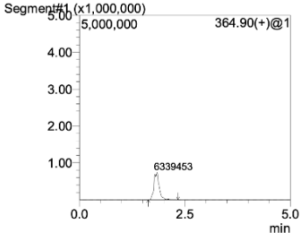<br>$[M+K^+]^+=365$
(R)<br>Lactose	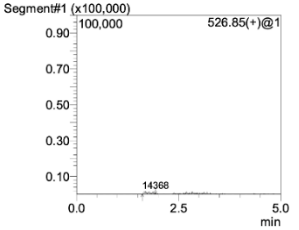<br>$[M+K^+]^+=527$	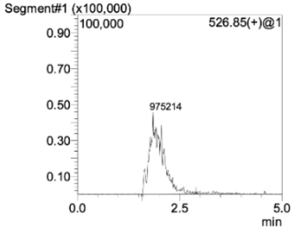<br>$[M+K^+]^+=527$	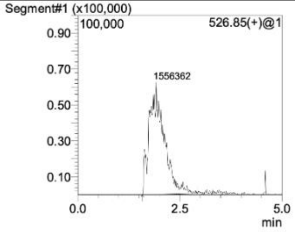<br>$[M+K^+]^+=527$	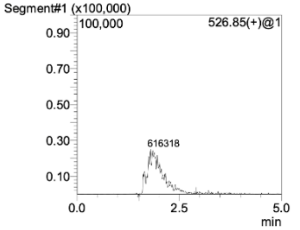<br>$[M+K^+]^+=527$	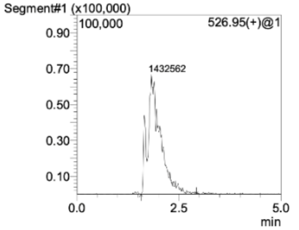<br>$[M+K^+]^+=527$
(S)<br>Maltose	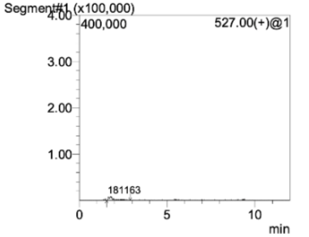<br>$[M+K^+]^+=527$	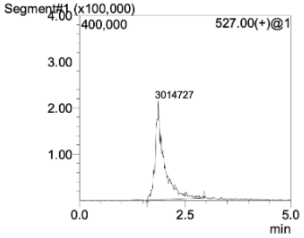<br>$[M+K^+]^+=527$	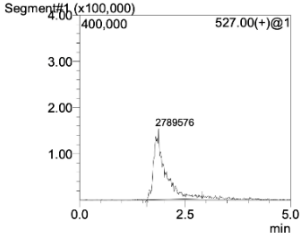<br>$[M+K^+]^+=527$	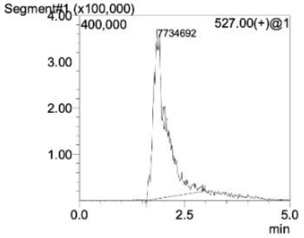<br>$[M+K^+]^+=527$	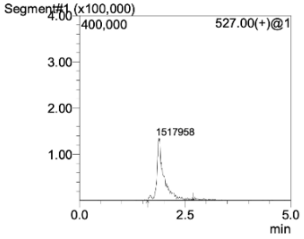<br>$[M+K^+]^+=527$
(T)<br>D-Mannose	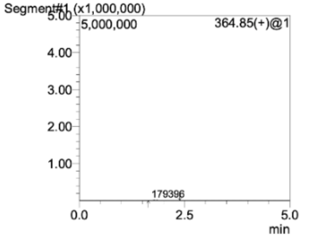<br>$[M+K^+]^+=365$	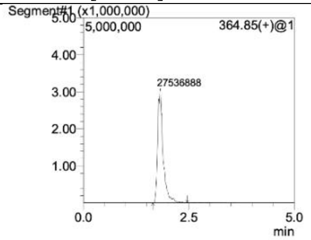<br>$[M+K^+]^+=365$	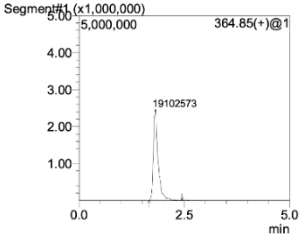<br>$[M+K^+]^+=365$	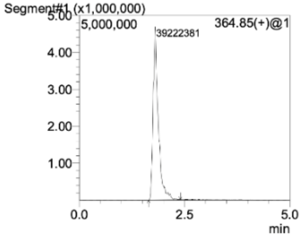<br>$[M+K^+]^+=365$	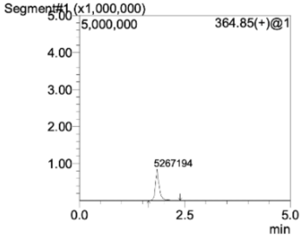<br>$[M+K^+]^+=365$

Acceptor molecule	Enzyme used for reaction and mass spectra of fucosylated compounds				
	Negative control	Fuc25A	Fuc25D	Fuc25E	Fuc25C
(U)<br>N-Acetyl glucosamine	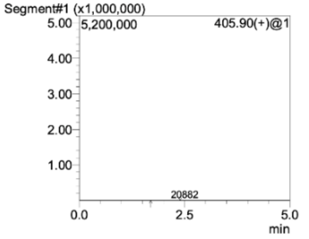<br>$[M+K^+]^+$=406	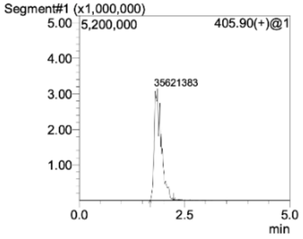<br>$[M+K^+]^+$=406	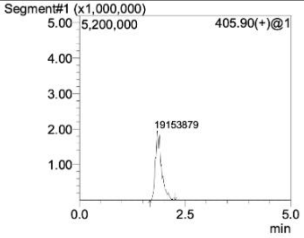<br>$[M+K^+]^+$=406	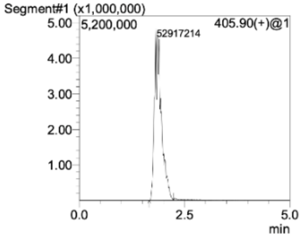<br>$[M+K^+]^+$=406	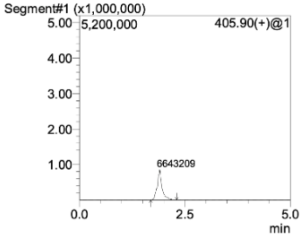<br>$[M+K^+]^+$=406
(V)<br>L-Rhamnose	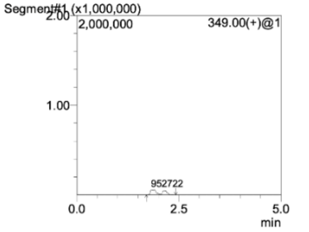<br>$[M+K^+]^+$=349	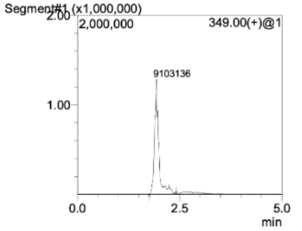<br>$[M+K^+]^+$=349	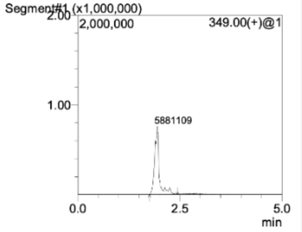<br>$[M+K^+]^+$=349	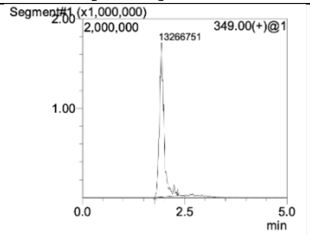<br>$[M+K^+]^+$=349	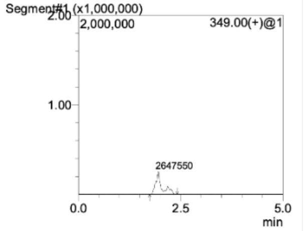<br>$[M+K^+]^+$=349
(Z)<br>D-Xylose	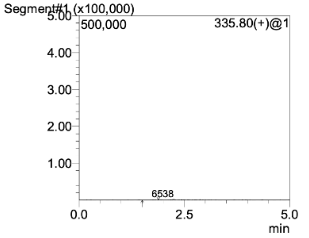<br>$[M+K^+]^+$=336	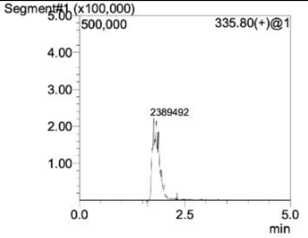<br>$[M+K^+]^+$=336	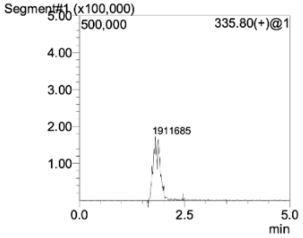<br>$[M+K^+]^+$=336	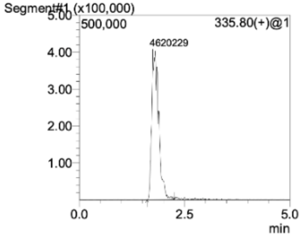<br>$[M+K^+]^+$=336	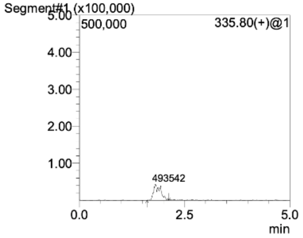<br>$[M+K^+]^+$=336

Fuc25A	Fuc25C	Fuc25D	Fuc25E

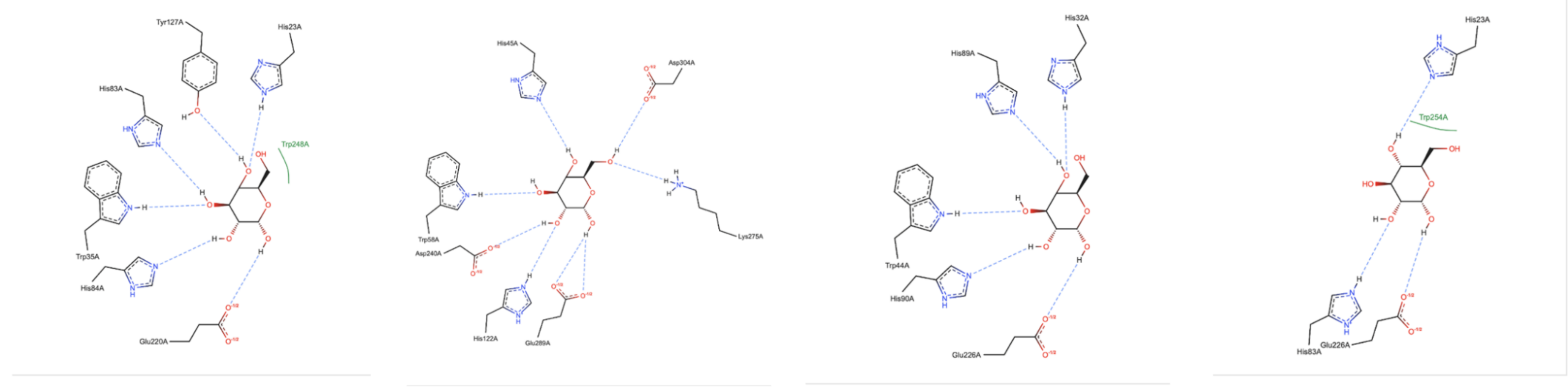

Figure S1 D-Glucose interactions with amino acid residues of the active site pocket determined by PoseEdit
