# Supplementary figures and images for: α-L-Fucosidases from an Alpaca Faeces Metagenome: Characterisation of Hydrolytic and Transfucosylation Potential
Source: Int J Mol Sci. 2024 Jan 9;25(2):809. doi: 10.3390/ijms25020809 (PMC10815079; doi:10.3390/ijms25020809)

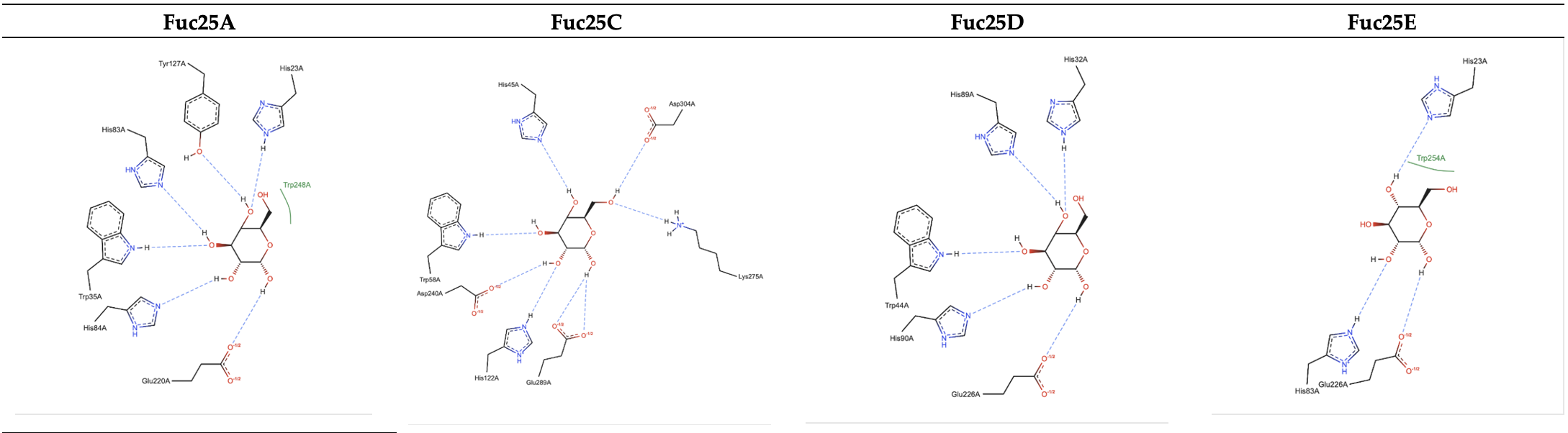

Supplement: Supplementary file 1 [file ijms-25-00809-s001.zip › Figure_S1.png]
